# Polyvinyl Alcohol (PVA)-Based Hydrogels: Recent Progress in Fabrication, Properties, and Multifunctional Applications

**DOI:** 10.3390/polym16192755

**Published:** 2024-09-29

**Authors:** Xiaoxu Liang, Hai-Jing Zhong, Hongyao Ding, Biao Yu, Xiao Ma, Xingyu Liu, Cheong-Meng Chong, Jingwei He

**Affiliations:** 1 School of Arts and Sciences, Guangzhou Maritime University, Guangzhou 510725, China; liangxxu@126.com (X.L.); max159159@163.com (X.M.); ixy820089031@foxmail.com (X.L.); 2 State Key Laboratory of Bioactive Molecules and Druggability Assessment, Jinan University, Guangzhou 510632, China; hjzhong@jnu.edu.cn; 3 College of Materials Science and Engineering, Nanjing Tech University, Nanjing 210009, China; hongyaoding@njtech.edu.cn; 4 School of Chemistry and Chemical Engineering, Lingnan Normal University, Zhanjiang 524048, China; y.biao@lingnan.edu.cn; 5 State Key Laboratory of Quality Research in Chinese Medicine, Institute of Chinese Medical Sciences, University of Macau, Macao 999078, China; 6 School of Materials Science and Engineering, South China University of Technology, Guangzhou 510641, China

**Keywords:** polyvinyl alcohol-based hydrogels, fabrication, biomedical, flexible device, environmental treatment

## Abstract

Polyvinyl alcohol (PVA)-based hydrogels have attracted significant attention due to their excellent biocompatibility, tunable mechanical properties, and ability to form stable three-dimensional networks. This comprehensive review explores the recent advancements in PVA-based hydrogels, focusing on their unique properties, fabrication strategies, and multifunctional applications. Firstly, it discusses various facile synthesis techniques, including freeze/thaw cycles, chemical cross-linking, and enhancement strategies, which have led to enhanced mechanical strength, elasticity, and responsiveness to external stimuli. These improvements have expanded the applicability of PVA-based hydrogels in critical areas such as biomedical, environmental treatment, flexible electronics, civil engineering, as well as other emerging applications. Additionally, the integration of smart functionalities, such as self-healing capabilities and multi-responsiveness, is also examined. Despite progress, challenges remain, including optimizing mechanical stability under varying conditions and addressing potential toxicity of chemical cross-linkers. The review concludes by outlining future perspectives, emphasizing the potential of PVA-based hydrogels in emerging fields like regenerative medicine, environmental sustainability, and advanced manufacturing. It underscores the importance of interdisciplinary collaboration in realizing the full potential of these versatile materials to address pressing societal challenges.

## 1. Introduction

Hydrogels are three-dimensional, hydrophilic polymer networks capable of absorbing large amounts of water or biological fluids [1,2,3]. These soft materials retain a distinct structure that allows for high water retention while maintaining mechanical integrity, providing both flexibility and the ability to mimic natural tissues, making them promising materials for various applications, such as drug delivery, tissue engineering, wearable sensors, flexible devices, actuators, etc. [4,5,6,7,8,9].

**Figure 1:**
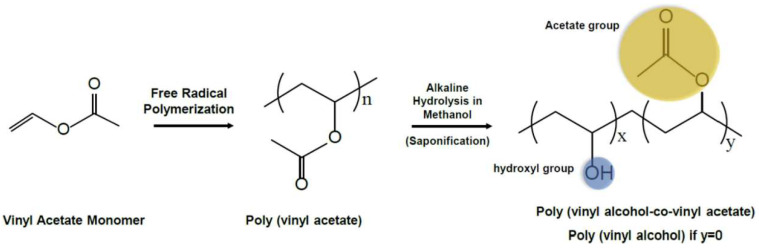
The synthesis route and chemical structure of polyvinyl alcohol (PVA). The partial hydrolysis of vinyl alcohol results in the formation of copolymers with vinyl acetate. The presence of the acetate groups imparts a lower tendency for crystallinity to the copolymers. x + y = n [10,11]. (Acetate group: yellow, hydroxyl group: blue).

Among the various materials employed in the fabrication of hydrogels, polyvinyl alcohol (PVA; the synthesis route and chemical structure of PVA are as shown in Figure 1) is of particular interest due to its unique characteristics, including excellent biocompatibility, water solubility, biodegradability, and the capacity to form stable networks via different cross-linking techniques. 

These qualities position PVA as a promising material in the realm of hydrogel innovation and research [12,13,14,15,16]. Hydrogels based on PVA exhibit numerous of outstanding features such as good biocompatibility, high water retention, good mechanical properties, and responsiveness to external stimuli [17]. These properties make them ideal candidates for a wide range of applications, including biomedicine, environmental remediation, and advanced technology development. In recent decades, there has been a notable increase in scientific research and publications dedicated to PVA-based hydrogels, highlighting their growing significance and potential applications [11,18].

The fabrication of PVA-based hydrogels has evolved significantly, encompassing both physical and chemical cross-linking methods [11,18,19,20]. Conventional techniques, such as freeze/thaw (F/T) cycles, continue to be of valuable, while innovative approaches, including electron beam irradiation (EBI) and the incorporation of multifunctional small molecules, have expanded the synthesis toolkit for hydrogels. These developments enable researchers to precisely modify the properties of PVA hydrogels, addressing challenges such as mechanical weakness and limited functionality [21,22].

Recent research has focused on enhancing the performance of PVA hydrogels through various strategies, including the development of composite materials, integration of nanostructures, and design of multi-network systems. These approaches have resulted in significant enhancements in mechanical properties, self-healing capabilities, electrical conductivity, and responsiveness to environmental stimuli. Consequently, the applications of PVA-based hydrogels are diverse, ranging from biomedical applications such as drug delivery systems and tissue engineering scaffolds to environmental applications like water purification and dust suppression. Moreover, these materials have shown potential in nascent fields such as flexible electronics, energy storage, and smart actuators, as well as civil engineering [20,23,24,25].

In view of the significant progress that has, this review aims to provide a comprehensive overview of the recent developments in PVA-based hydrogels. It will focus on the fabrication methods employed, the enhancements made to their properties, and the various applications in which they are used. Finally, the review will examine the remaining challenges in the field and discuss prospects for the development and application of these interesting materials. It is expected that this review will help researchers gain a thorough understanding of the current state of the field and inspire future innovations in PVA-based hydrogels.

## 2. Fabrication and Enhancement of PVA-Based Hydrogels

The preparation method of PVA-based hydrogel can be broadly classified into two primary categories: physical cross-linking and chemical cross-linking. Due to the inherent water solubility of PVA, cross-linking is necessary to create hydrogels that are suitable for various applications. These cross-links, whether physical or chemical, impart crucial structural stability to the hydrogel, allowing for it to swell appropriately when exposed to water or biological fluids. The degree of cross-linking has a significant impact on the fluid absorption capacity of hydrogels, which in turn affects their physical, chemical, and diffusional properties. This ultimately determines the suitability of hydrogels for practical applications [26,27,28].

### 2.1. Physical Cross-Linking Method of PVA-Based Hydrogels

**Figure 2:**
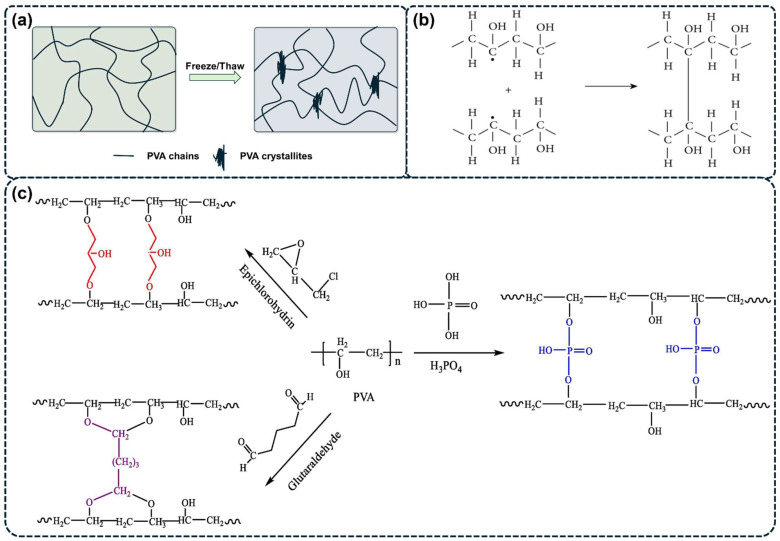
Schematic illustration of the gelation process of PVA hydrogels. (**a**) The fabrication of PVA hydrogels via freeze/thaw (F/T) process [17]. (**b**) Irradiation cross-linking process of PVA hydrogels [29]. (**c**) Chemical reagent cross-linking process of PVA hydrogels [30].

The physical cross-linking method primarily involves the repeated F/T cycles (as shown in Figure 2a).

The regular linear structure of PVA and its abundant hydroxyl groups facilitate the formation of ordered arrays through intramolecular hydrogen bonding, resulting in a crystalline cross-linking network and subsequent hydrogel formation after multiple F/T cycles. An F/T cycle typically involves freezing an aqueous PVA solution at concentrations ranging −10 °C to −40 °C for about 12 to 24 h, followed by thawing it at room temperature (25 °C) for 1 to 3 h [11,26,27,31]. These physically cross-linked PVA hydrogels are preferred for various applications (e.g., tissue engineering, targeted drug delivery, wound dressing) due to their high purity, facile gelation process under mild conditions, and the absence external cross-linking agents [32,33,34]. They exhibit remarkable mechanical strength, good elasticity, and high viscosity. Insoluble in water at room temperature, these hydrogels have a smooth surface, high water content, and excellent mechanical properties; however, they are sensitive to temperature changes and have poor transparency [35].

The properties of PVA hydrogels prepared through the F/T process are significantly influenced by gelation parameters, including duration, temperature, and the number of F/T cycles. Additional factors, such as molecular weight, concentration, and degree of hydrolysis of PVA also play important roles [36]. Key indicators of hydrogel quality include hydrogel quality, including strength and swelling capacity; higher gelation points generally result in stronger hydrogels with reduced water absorption. Adjusting freezing temperatures and durations can fine-tune the properties of hydrogels [37,38]. For instance, slower rates of temperature change during both freezing and thawing steps can enhance PVA crystallization. Ideally, the thawing rate should be less than 10 °C/min, with some studies suggesting rates as low as 0.2 °C/min [37]. Furthermore, fully hydrolyzed (98.5%) PVA hydrogels exhibit higher strength compared to partially hydrolyzed (88%) PVA hydrogels [36]. Additionally, increasing PVA concentration generally enhances the mechanical properties and stability of the hydrogels, but it also affects other characteristics such as pore size and water uptake. A minimum concentration of 10 *wt%* is typically used in the literature, depending on the specific requirements of the intended application [26,27].

The transparency of PVA hydrogel is a complex function influenced by polymer concentration, the number of freeze/thaw cycles, average molecular weight, and degree of hydrolysis. Typically, repeated freeze/thaw treatments lead to randomly distributed heterogeneity, causing the hydrogel to become cloudy or opaque. High transparency can be achieved by forming small fibril-like crystallites and pores smaller than 3 µm, which enhance light transmittance [33,34,39]. Some transparent F/T PVA hydrogels have been developed by used dimethyl sulfoxide (DMSO) as a co-solvent, with a process involving freezing at 0 °C for 8 h, followed by 16 h of thawing at room temperature or 37 °C with the process repeated for 15 to 45 cycles [39,40,41]. During the fabrication, DMSO formed hydrogen bonds with PVA, altering its crystallization by inhibiting two-dimensional crystal growth. This suppression reduced crystal grain size, enhancing light transmission and improving transparency [40]. This method, however, can be extremely time-consuming and involves the use of a highly toxic chemical solvent [39,40,41]. To address these issues, Gupta et al. developed an innovative method for synthesizing transparent PVA hydrogels without using toxic solvents like DMSO. The authors demonstrated that stable, transparent PVA hydrogels can be produced using only water as a solvent and employing a freeze-thaw cycle between 0 °C and room temperature, rather than the conventional method of cycling down to −20 °C. This milder temperature range is more suitable for potential cell encapsulation applications. Results showed that increasing PVA concentration leads to higher crystallinity, reduced porosity, and decreased water absorption. This work represents a significant advancement in PVA hydrogel synthesis, offering a less toxic and potentially more cell-friendly approach while maintaining transparency [33].

As research and technology continue to advance, investigators have developed PVA hydrogels with enhanced mechanical properties through improved F/T methods, such as F/T under drawing [42] and directional F/T (high tensile strengths of 0.3–1.2 MPa, medium moduli of 0.03–0.10 MPa, and high fracture energies of 160–420 J/m^2^) [43,44].

The traditional method for the fabrication of PVA-based hydrogels involves a repeated F/T method in which freezing temperature and longer time are required. However, hydrogel’s relatively poor mechanical strength continues to limit its application in various fields. Additionally, elevated temperatures can disrupt hydrogen bonds and microcrystalline regions, causing the physical cross-linked PVA hydrogel to melt and revert to a solution state, requiring further improvement [45].

### 2.2. Irradiation Cross-Linking Methods

Chemical cross-linking of PVA occurs through reactions between its hydroxyl groups and functional groups on other molecules. This process can be divided into radiation cross-linking (as shown in Figure 2b) [46,47,48,49] and chemical reagent cross-linking (as shown in Figure 2c) [18,20,28].

#### 2.2.1. Irradiation Cross-Linking

The radiation technique is an effective tool for the improving or modifying polymer materials through cross-linking, grafting, or degradation [50]. High-energy irradiation including gamma radiation [46], EBI [47], X-rays [48], etc., induces the formation of free radicals on PVA chains, leading to the creation of structurally stable covalent bonds. This process results in a safe and non-toxic three-dimensional network structure [24,49]. For instance, Hiep et al. successfully fabricated CS/PVA/Silver nanoparticles (CPA) hydrogels with microwave assistance to reduce silver ions to silver nanoparticles in situ and to cross-link CS with PVA [49].

Gamma radiation-induced cross-linking is recognized as a suitable and convenient method for polymer network formation. The degree of cross-linking and structural stability of the hydrogel are influenced by the irradiation dose and time. For instance, Salmawi et al. investigated the impact of gamma irradiation doses on PVA-based hydrogels. Mixture of PVA/CS in different ratios were exposed to gamma irradiation doses of 20, 30, and 50 kGy to assess the effect on the blend’s physical properties. Results revealed that the gel fraction increases with higher irradiation doses and greater PVA concentration in the blend [46].

EBI also plays a crucial role in the preparation of these hydrogels, due to their rapid (approximately 30 s) in situ chemical cross-linking of PVA, free radical generation, and environmentally friendly nature. Additionally, EBI can regulate the structure and cross-linking degree of the hydrogels by controlling irradiation conditions to achieve desired properties [51,52]. For example, Hao et al. fabricated ionogels using in situ EBI to cross-link PVA and PVP. This innovative method allows for precise control of the polymer network’s gradient structure, resulting in ionogels with remarkable mechanical properties, including high stretchability (>1000%), exceptional toughness (100 MJ/m^3^), and a distinctive gradient modulus. The ionogels exhibit an ultra-fast response time (60 ms) comparable to skin, an incredibly low detection limit (1 kPa), and an exceptionally wide detection range (1 kPa–1 MPa) [47].

The use of irradiation technology represents a significant advancement in the preparation of cross-linked polymers. Irradiation allows for the production of final products that are free from impurities. However, this cross-linking method still faces limitations. One of the drawbacks is the potential to damage the biological activity of tissues and cells during the irradiation process. It is essential to load the hydrogels with biologically active materials after their irradiation cross-linking, as the radicals formed during irradiation can damage these substances. Furthermore, because the cross-links forming in irradiated PVA are carbon–carbon (C-C) bonds, these hydrogels are not biodegradable [47,49,50].

#### 2.2.2. Chemical Reagent Cross-Linking

Chemical reagent cross-linking is one of the earliest strategies for fabricating PVA hydrogels [53,54,55,56,57]. In this process, bifunctional chemical cross-linkers are introduced into the PVA solution. These cross-linkers react with the hydroxyl groups on PVA and then form a covalently bonded cross-linked network, resulting in a chemically cross-linked PVA hydrogel. Commonly used chemical cross-linkers for PVA include aldehydes, anhydrides, isocyanates, borates, and their derivatives [18,20,28].

The efficiency of the cross-linking reaction and the resulting properties of the hydrogel are influenced by factors such as pH, temperature, and reaction duration. By manipulating the degree of cross-linking—through the amount of cross-linking agent used and the length of the reaction time—it is possible to tailor the hydrogel’s mechanical strength, pore size, water absorption, and other characteristics [28,58]. For instance, Shagholani et al. investigated the influence of cross-linking agents (glutaraldehyde (GA) and ammonium persulfate (APS)) on properties of PVA-based hydrogels. GA cross-linked hydrogels demonstrated a higher drug loading capacity (10%), greater pH sensitivity, and faster drug release, especially in acidic conditions, compared to APS cross-linked hydrogels, which had a drug loading capacity of 5.9%. GA cross-linking forms flexible bridges between polymers, enhancing their ability to swell, especially in acidic environments. In contrast, APS uses a radical mechanism to tightly cross-link polymers, limiting their swelling capacity [28].

However, the majority of chemical cross-linkers currently in use tend to be biotoxic and difficult to remove. These cross-linkers can significantly harm tissue cells and trigger inflammation post-implantation, thereby severely compromising the biocompatibility of these PVA-based hydrogels. As a result, there is a growing interest in developing alternative cross-linking methods that are both effective and biocompatible [20].

### 2.3. Enhancement of PVA-Based Hydrogels

Despite undergoing various cross-linking processes, numerous PVA-based hydrogels continue to exhibit limited mechanical strength, restricting their further applications. Consequently, numerous researchers are exploring methods to enhance the mechanical properties of polyvinyl alcohol hydrogels to broaden their potential applications [48,59,60,61,62,63].

Molecular weight is crucial for the properties of polymeric materials. Generally, low molecular weight PVA does not form stable gels, but higher molecular weight PVA contributes to greater tensile strength and elasticity of the hydrogel. This improvement is attributed to the longer polymer chains, which can become entangled or cross-linked, forming a more stable network capable of withstanding mechanical stress [53,64,65,66]. For instance, Rong et al. investigated PVA with molecular weights of 30, 70, and 130 kDa after a single F/T cycle and showed that only the 130 kDa sample gelled, mainly due to the increasing cross-linking point as the molecular weight of the PVA increased [67]. Similarly, Xue et al. systematically investigated the relationship between the molecular weight of PVA and the mechanical properties of PVA–graphene oxide (GO) composite hydrogels. Results showed that higher molecular weight PVA led to improved mechanical properties of the hydrogels, with increased storage and loss moduli indicating enhanced elasticity and stability. The critical gel concentration required for hydrogel formation decreased with increasing PVA molecular weight, allowing for gel formation at lower PVA concentrations. Additionally, higher molecular weight PVA resulted in denser network structures and stronger hydrogen bonding with GO sheets, enhancing interfacial adhesion and load transfer [64].

PVA chains can form entanglements in aqueous systems in the presence of salt ions, a phenomenon known as the Hofmeister effect. Recent studies have demonstrated that this effect is a valuable method for tuning the mechanical properties of PVA hydrogels [59,60,61,68,69]. For instance, inspired by cell dehydration in electrolyte solutions, Miao et al. used a simple salting out method using Zn^2+^ to induce both multi-cross-linking and salting-out effects in the hydrogels. By adjusting the degree of salting-out, the researchers could regulate the structure and tribological properties of the hydrogels, leading to a structurally stable hydrogel with excellent load bearing capacity and superior lubrication performance [59]. Hua et al. have innovated a pioneering fabrication strategy that integrates directional freeze-casting and subsequent salting-out treatment to enhance the mechanical properties of PVA hydrogels. The directional freeze-casting process meticulously controls the solidification front of a solvent, typically water, within the hydrogel precursor solution. This precision dictates the alignment and orientation of pores by directing the growth of ice crystals, effectively forming a template for microporous structures. Followed by this, the salting-out treatment is applied, introducing a high concentration of salt to the hydrogel, which induces phase separation. This occurs as salt molecules outcompete water molecules in associating with polymer chains, leading to dehydration. Consequently, this separation fosters the formation of interconnected nanofibril meshes within the hydrogel matrix. This dual-process method synergistically constructs multiscale hierarchical structures within the PVA hydrogels, extending from aligned micropore walls to interconnected nanofibril meshes. Such a setup significantly boosts the strength, toughness, stretchability, and fatigue resistance of the hydrogels compared to conventional forms. Impressively, the resulting hydrogels display outstanding mechanical properties, including a high ultimate stress of 23.5 ± 2.7 MPa, a large strain capacity of 2900 ± 450%, extensive toughness measured at 210 ± 13 MJ.m^−3^, and superior fatigue resistance. These mechanical enhancements are achieved while preserving a high-water content, ranging from 70% to 95%, matching or exceeding those found in natural tendons and cutting-edge hydrogels. This remarkable combination of properties makes these hydrogels particularly well-suited for challenging applications in medical devices, robotics, and energy systems [61].

**Figure 3:**
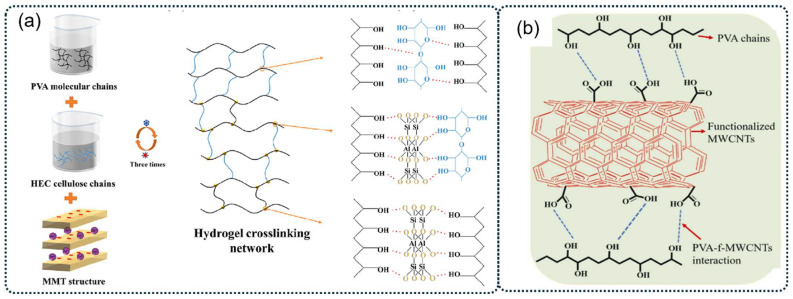
(**a**) Synthesis and structure diagram of *P*–H3%-M2% hydrogel [63]. (**b**) Schematic representation showing the intermolecular H-bond between PVA chains and -COOH functional groups of f-MWCNTs [71].

The incorporation of inorganic or organic additives can enhance the cross-linking degree of PVA molecular chains and improve the overall performance of PVA hydrogels [63,70]. For instance, Ma et al. created a novel composite PVA hydrogel electrolyte by integrating hydroxyethyl cellulose (HEC) and montmorillonite (MMT) into the PVA hydrogel matrix (as illustrated in Figure 3a). The synergistic cross-linking effect resulted in a dual cross-linked network structure. The composite hydrogels demonstrated enhanced mechanical properties, with the *P*–H_3%_-M_2%_ hydrogel composed of 3 *wt%* HEC and 2 *wt%* MMT showing significant increases in tensile strength (242.6%), elongation at break (78.6%), and compressive strength (970.1%) when compared to pure PVA hydrogel [63]. Swati Sharma et al. first reported a PVA/functionalized multiwalled carbon nanotube (PVA/f-MWCNTs) nanocomposite hydrogel with enhanced dielectric properties using a one-step high-shear mixing technique followed by natural cooling at room temperature. The stable hydrogel network was formed through intermolecular hydrogen bonding between the -OH groups of PVA and -COOH groups of f-MWCNTs (as shown in Figure 3b). The nanocomposite hydrogel demonstrated approximately five- and sixfold increases in modulus and hardness, respectively, compared to pristine PVA hydrogel. This physical method offers several advantages over other techniques, including no need for chemical cross-linkers or freezing temperatures, and a shorter required gelation time [71].

To address the longstanding challenge of developing PVA hydrogels with robust mechanical properties in diverse environments, Wan et al. combined a monomer-induced phase separation technique with thermal annealing to create a PVA/Poly(2-methoxyethyl acrylate) (PVA/PMEA) composite hydrogel with multiscale nanostructures that exhibits exceptional strength and toughness in both aqueous (including harsh acidic, alkaline, and saline conditions) and atmospheric environments. The hydrogel showed a breaking strength of up to 34.8 MPa and toughness of up to 214.2 MJ/m^3^ in its hydrated state. Meanwhile, the dehydrated plastic form achieved even higher values of 65.4 MPa and 430.9 MJ.m^−3^, respectively. This study offered a promising strategy for engineering high-performance, environmentally adaptable polymer materials for complex load-bearing applications [62].

Hydrogels can be further enhanced by integrating non-covalent interactions with intricately designed network structures, a technique that efficiently dissipates energy through the reversible breaking of non-covalent bonds. Synergistic interactions are proposed as key strategies to enhance toughness. In this approach, weaker reversible interactions dissipate energy when ruptured, while stronger interactions provide structural stability [25,31,72,73]. For instance, Zhu et al. introduced an innovative method to simultaneously enhance the strength, toughness, and stretchability of hydrogels by incorporating multiple non-covalent interactions within a multi-network structure. This synergistic integration involves hydrogen bonding, chain entanglements, PVA crystallites, and metal ion coordination within interpenetrating networks of polyacrylamide (PAAm), PVA, and alginate. The stepwise enhancement in mechanical properties from single network to double network and triple network hydrogels demonstrated the effectiveness of combining multiple non-covalent interactions and network structures in designing tough and stretchable hydrogels. Such a strategy enables hierarchical energy dissipation mechanisms, resulting in hydrogels with excellent mechanical properties, including a tensile strength of up to 2.82 MPa and a fracture energy of 11.50 MJ/m^3^, while maintaining high stretchability [31]. Han et al. presented a novel triple network hydrogel sensor based on SA, PVA, and PAM with KCl; this hydrogel combines the mechanical robustness of PAM with the flexibility of sodium alginate/PVA (SA/PVA), resulting in excellent stretchability (1250%), high toughness (4.8 MJ/m^3^), and self-healing properties [25].

With further exploration into novel fabrication technologies, the use of natural and non-toxic materials, and the incorporation of nanocomposites, PVA hydrogels are poised to overcome current limitations and achieve new levels of functionality and adaptability. These advancements are anticipated to propel the capabilities of hydrogels, offering solutions that are not only effective but also safe for clinical and industrial use.

## 3. Properties and Applications of PVA-Based Hydrogels

PVA-based hydrogels are becoming increasingly prominent due to their functional versatility, which stems from a unique three-dimensional network structure characterized by high water content and exceptional water absorption capacity. This structure not only enhances flexibility and biocompatibility but also allows for easy processing and molding into varied shapes due to the hydrogels’ chemical stability and resistance to biological aging [17,74,75,76]. The hydrophilic nature of PVA enables hydrogen bond formation with water and other solvents, enhancing permeability and facilitating efficient substance transfer—a crucial attribute for controlled release applications [77]. Moreover, the low coefficient of friction makes them suitable for applications requiring smooth, low-resistance surfaces [78].

Despite these advantages, PVA-based hydrogels face challenges such as relatively weak mechanical strength and limited cell adhesion, which can hinder their use in tissue engineering. To overcome these limitations, modifications like component incorporation and cross-linking techniques are employed to improve mechanical properties [48,79,80]. Furthermore, PVA-based hydrogels offer significant potential for functionalization, enabling enhancements such as shape memory capabilities, conductivity, stimuli responsiveness, and dye adsorption capacity [17,80,81,82]. Their compatibility with advanced fabricate technologies further extends their application potential, allowing for the fabrication of complex structures suitable for drug delivery systems, wound dressings, artificial tissues and organs, intelligent materials, environmental treatment, and civil engineering [82,83,84,85]. Continued research is crucial for improving long-term biocompatibility and multifunctionality, thereby expanding the application potential of PVA-based hydrogels in emerging fields. This ongoing innovation is a priority in the materials science community and is explored in the following sections [10,86,87,88].

### 3.1. Biomedical Application of PVA-Based Hydrogels

As one of the few FDA-approved (U.S. Food and Drug Administration) excipients, PVA-based hydrogels are widely used in biomedical applications, including drug delivery systems, wound dressings, and tissue engineering [10,15,74,75,76,89,90]. Recent advances in PVA-based hydrogels in biomedical applications will be discussed in this section, and examples of representative PVA-based hydrogels are summarized in Table 1.

#### 3.1.1. Drug Delivery Systems

PVA-based hydrogels are considered a stable matrix for the drug delivery due to their non-toxic nature, biocompatibility, and gelling properties [89,90]. These hydrogels can encapsulate various of therapeutic agents, such as small molecules, proteins, and peptides, facilitating controlled release profiles that enhance therapeutic efficacy while minimizing side effects. However, the application of conventional pure PVA hydrogels in drug delivery systems is limited by their undesirable opaque appearance, low swelling capacity, and inert reactivity. It is expected that combining PVA with other components will yield novel hydrogels with improved structure and enhanced drug loading/release performance can be developed [86,91,92,93,94,95]. For instance, Fabián Martínez-Gómez et al. developed stable SA/PVA hydrogels cross-linked by hydrogen bonds, demonstrating excellent swelling capacity, pH and temperature sensitivity, controlled drug release, and tunable properties by controlling polymer ratios and preparation conditions. The hydrogels achieved up to 55% metformin release from 1.0:1.0 *w/v* SA/PVA hydrogels at 37 °C after 72 h, suggesting their controlled release properties in the intestinal tract. Such mild fabrication strategies are suited to drugs with diverse physicochemical properties, including enzymes, peptides, and proteins [86].

Conventional drug delivery modes include systemic and localized. Localized drug delivery, however, enables direct application to specific sites, enhancing drug utilization and reducing side effects [96]. For example, injectable hydrogels have gained significant attention in the treatment of periodontitis due to their ease of administration, biocompatibility, and ability to provide controlled drug release. Qiu et al. prepared a dynamic cross-linked hydrogel (HOBP) with hydroxypropyl chitosan (HPCS) and PVA as substrates, with borax and oxidized sodium alginate (OSA) as cross-linkers. The combination of injectability, self-healing, tunable mechanics, controlled drug release, antibacterial effects, and biocompatibility makes this hydrogel system promising for localized drug delivery in complex environments such as the oral cavity for periodontitis treatment [77].

Integrating GO into hydrogels can not only improve the mechanical properties and electrical conductivity of PVA hydrogels, but also enhance its potential application in drug delivery [79,93,97]. For instance, GO/PVA composite hydrogel is biocompatible and pH-sensitive, while 84% of the vitamin B12 molecules can diffuse from the hydrogel into the pH-neutral PBS solution within 42 h. The incorporation of GO creates an extended pathway for drug diffusion, prolonging release time compared to the pure PVA hydrogel. In addition, GO reduces the initial burst release of the drug due to the electrostatic interactions or physical entrapment, including hydrogen bonding with drug molecules, resulting in a more controlled and sustained drug release profile [94]. Xiong et al. constructed a composite hydrogel with PVA, CS, and GO as the conductivity patch and loaded with sodium fluorescein (NaFL) as the drug model. They examined the combined effects of drug release and tissue repair under electrical stimulation, where low-voltage direct current pulses enhanced cell growth, migration, and the release of vascular endothelial growth factor (VEGF) and basic fibroblast growth factor (FGF). Direct current stimulation increased fluorescein sodium salt permeability in the hydrogel, suggesting that carbohydrate polymer hydrogels could function as controlled drug carriers, with electrical stimulation providing new opportunities for functional drug delivery and transdermal therapy [93].

These advancements in PVA-based hydrogel formulations and their ability to facilitate controlled drug release underscore their potential as effective carriers in therapeutic applications, leading the way for innovative strategies in drug delivery and personalized medicine.

#### 3.1.2. Wound Dressing

PVA-based hydrogels have garnered significant attention for wound healing due to their excellent biocompatibility, biodegradability, and non-carcinogenicity, making them ideal candidates for advanced wound dressing materials. These hydrogels provide a moist environment that is crucial for promoting cell migration and tissue regeneration, essential for effective wound healing [74,75,76]. However, traditional pure PVA hydrogels often exhibit limited elasticity and hydrophilicity, which can restrict their use as standalone wound dressings. To overcome these limitations, researchers have explored the integration of various bioactive materials, such as CS, hyaluronic acid, and collagen, into PVA-based hydrogels. This incorporation enhances the mechanical properties and hydrophilicity of the dressings and introduces bioactive functionalities that promote cellular activities and accelerate healing [98,99,100,101,102,103]. 

For instance, combing PVA with CS yields hydrogels with improved antibacterial properties, crucial for preventing infections at wound sites. Incorporating nanoparticles like silver or GO into PVA hydrogels has further enhanced their antimicrobial efficacy while also improving mechanical strength and flexibility. Recent advancements have led to the development of smart wound dressings that can respond to environmental stimuli, such as pH or temperature changes, allowing for controlled drug release and real-time monitoring of wound conditions. These innovative dressings can also be designed to provide sustained release of therapeutic agents, such as anti-inflammatory drugs or growth factors, directly at the wound site, thereby enhancing healing performance [87,104]. Lan et al. successfully synthesized rGO-PDA@ZIF-8/PVA/CS composite hydrogels using bidirectional freezing and phase separation techniques. These hydrogels, with a directional macroporous structure, exhibited remarkable antibacterial efficacy against both *E. coli* and *S. aureus* (99.1% and 99.0%, respectively) under the synergistic effect of intrinsic antibacterial activity and photothermal antibacterial. Furthermore, the hydrogels exhibited significant potential in promoting wound healing, making them promising candidates for advanced wound management applications [87]. Zhao et al. engineered a collagen/PVA hydrogel containing BMn (BMn@G), designed to deliver anti-inflammatory and pro-healing benefits during wound healing. The implementation of F/T collagen/PVA hydrogels significantly improves the encapsulation efficiency of bilirubin/morin nanoparticles within the matrix, thereby accelerating healing [104].

As research progresses, the potential for PVA-based hydrogels in wound dressing is expected to grow, creating opportunities for developing new and innovative therapeutic strategies that address the complexities of wound care.

#### 3.1.3. Tissue Engineering

PVA-based hydrogels have been extensively studied as a highly promising material in tissue engineering, owing to their excellent biocompatibility, tunable mechanical properties, and ability to mimic the natural extracellular matrix (ECM) of biological tissues. These hydrogels provide a supportive environment for cell adhesion, proliferation, and differentiation, making them ideal scaffolds for various tissue engineering applications, including cartilage, bone, and vascular tissues. Additionally, the unique slippery characteristics of PVA hydrogels closely resemble those of soft biological tissues, making them particularly advantageous for applications such as artificial joints and contact lenses [78,82,88,105,106,107].

Natural biological tissues, like ligaments, possess anisotropic structures at varying scales and retain high water content while maintaining significant strength and flexibility. However, conventional hydrogels are generally isotropic and homogeneous, lacking the strength and fatigue resistance required at high water content in human tissues. Consequently, extensive research has focused on developing biomimetic PVA-based hydrogels with anisotropic structures and enhanced mechanical properties through methods including mechanical stretching, directional freeze-casting, and the incorporation of additional fillers [24,35,88,105]. For instance, Han et al. designed and developed a tough, fatigue-resistant PVA/carbon nanotubes (CNT) hydrogels as artificial ligaments through a freezing-casting-assisted annealing and salting-out (FCAS) strategy [88]. This innovative approach endowed the hydrogels with low hysteresis, good biocompatibility, excellent mechanical properties (strength of 4.5 MPa and fatigue threshold of 1.5 kJ/m^2^), and a high water content of 79.5%. The exceptionally tough hydrogel demonstrates significant potential as an advanced artificial tissue, capable of replicating the structure and function of human tissue [88]. 

Recent advancements have also explored incorporating conductive materials, including CNT or graphene, into PVA hydrogels to create electrically active scaffolds that can enhance cellular responses and promote tissue integration. These conductive hydrogels are particularly relevant for neural tissue engineering application, where electrical stimulation can facilitate nerve regeneration and improve functional recovery. Furthermore, the development of smart PVA-based hydrogels that respond to external stimuli, such as temperature or pH changes, offers exciting possibilities for creating dynamic scaffolds that can adapt to the physiological environment of the tissue [93,105,108]. For instance, Li et al. fabricated an anisotropic PVA/CNT hydrogel by using a drying and rehydration approach to densify the polymer network. The resultant hydrogel demonstrated excellent anti-swelling ability (<3%), high tensile strength (3.71 MPa), and toughness (9.86 MJ/m^3^) upon hydration, at a tendon-like water content of 72.5 *wt%*. In addition, these hydrogels can be implanted as tendon substitutes in Sprague–Dawley rats with tendon defects, thereby aiding in dysfunctional tissue therapy and rehabilitation [105].

A prevalent, chronic degenerative disease, osteoarthritis affects millions worldwide, characterized by cartilage destruction and inflammatory reactions. A key challenge in treating osteoarthritic joints is managing excessive reactive oxygen species (ROS), which exacerbate inflammation and tissue damage. However, current treatments often fall short in terms of efficacy or safety [106,109,110]. In a significant advancement, Lei et al. reported a novel hydrogel (oHA-PBA-PVA) formulated from 3-aminophenylboronic acid-modified hyaluronic acid, cross-linked with PVA. This hydrogel possesses ROS-scavenging capabilities and provides joint lubrication. Its innovative structure, characterized by dual dynamic covalent bonds (Schiff base and phenylboronic ester), ensures properties such as injectability, self-healing, and ROS-responsive degradation. The hydrogel demonstrated excellent biocompatibility and sustained release of hyaluronic acid, along with significant anti-inflammatory effects, both in vitro and in vivo. Remarkably, it outperformed conventional hyaluronic acid treatments, achieving better cartilage repair and reduced inflammatory markers in osteoarthritic mouse models, without the addition of pharmaceuticals. This multifunctional design suggests a promising strategy for osteoarthritis management, potentially reducing the need for frequent injections while providing sustained therapeutic benefits [106].

**Figure 4:**
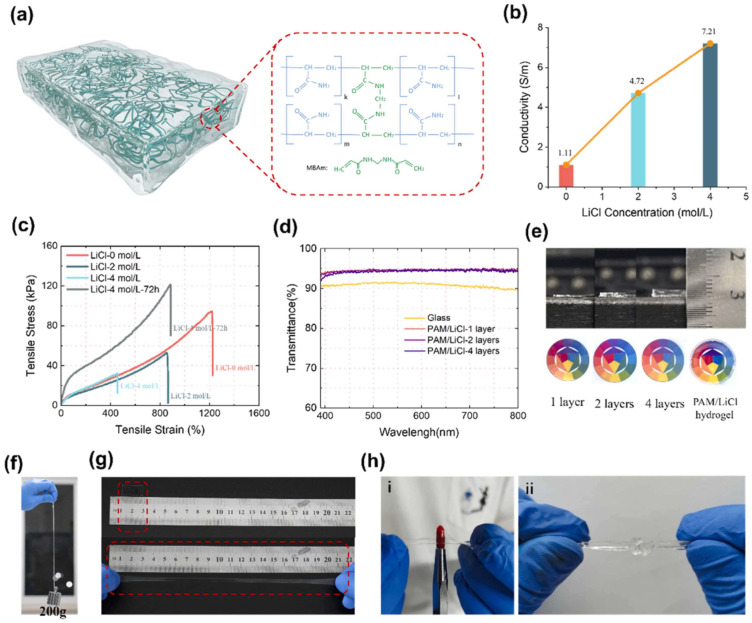
Characteristics of hydrogels at different ion concentrations. (**a**) Schematic of the hydrogel structure and molecular structure of a covalently cross-linked polymer chain. (**b**) Conductivity, (**c**) tensile curves, and (**d**) transmittance of visible light of PAM/LiCl hydrogels. (**e**) Optical image of PAM/LiCl hydrogels with different layers. (**f**) The hydrogel lifts an object with 200 g. (**g**) Photographs of the initial (stretch λ = 1) and stretched (λ = 12) state of the hydrogel. (**h**) Mechanical stretch of PAM/LiCl hydrogels with poking and in a knot [111].

Existing PVA-based hydrogels often struggle to simultaneously achieve high mechanical strength, optical transparency, and lubricity [68,78]. To address this challenge, Liu et al. developed a novel fabrication strategy termed “salting-out-after-syneresis” to create PVA-based hydrogels with an unprecedented combination of properties. The fabrication involved first densifying the PVA network through syneresis, followed by salting-out to induce rapid phase separation and crystallization. The resulting hydrogels exhibit remarkable transparency (up to 98% in the visible region), excellent lubricity (coefficient of friction as low as 0.0081), and superior mechanical properties (strength of 26.72 MPa, modulus of 6.66 MPa, and toughness of 55.21 MJ/m^3^). This approach demonstrated potential for applications in biomedicine and wearable devices, such as contact lenses with enhanced comfort and functionality [78]. Building upon the advantages of PVA-based hydrogels, Niu et al. achieved another milestone by leveraging the highly transparent (95% light transmittance) and conductive properties of PVA-based hydrogels (as shown in Figure 4). They successfully transferred high-performance, small-sized (94 μm) micro-LED arrays onto the PVA hydrogel substrate with high precision and minimal damage, achieving a resolution of 254 PPI. The hydrogel’s biocompatibility enabled the creation of an ultra-thin (200 μm) contact lens display, demonstrating promising potential for advanced human–computer interaction and augmented reality applications. This work represents a significant advancement in flexible, customizable, and implantable display technologies, paving the way for next-generation visual interfaces in various fields, including wearable electronics and biomedical devices [111].

**Table 1:** Summary of representative PVA-based hydrogels in biomedical applications.

Hydrogel System	Fabrication Method	Results	Limitation	Application	Ref.
SA/PVA hydrogel	F/T cycle	1. High swelling ratios achieved (up to 20 g/g in DI water). 2. Low drug release at pH 1.2; highest release (55%) at pH 8.0 after 6 h. 3. Release kinetics indicated non-Fickian diffusion mechanism. 4. Good mechanical properties and biocompatibility.	1. Decreased stability at pH 8.0 after 5–6 h. 2. Only in vitro studies conducted.	Drug delivery carriers	[86]
β-cyclodextrin/CS-based (PVA-co-acrylic acid) hydrogels	Free radical grafting technique	1. pH-sensitive swelling and drug release, peaking at pH 7.4. 2. Enhanced bioavailability of gallic acid with higher plasma concentrations than free drug solutions. 3. Good antioxidant and antibacterial properties.	1. Tested only with gallic acid. 2. Long-term stability not evaluated.	Controlled drug delivery systems	[92]
Hydroxypropyl chitosan/PVA hydrogel (HOBP)	HPCS and PVA cross-linked with borax and OSA.	1. Excellent injectability and self-healing properties. 2. High antimicrobial efficacy (*E. coli*: 86.18%, *S. aureus*: 85.69%). 3. High biocompatibility (cell viability >80%). 4. Favorable slow-release drug performance (168 h).	1. Long-term in vivo stability and degradation not extensively studied. 2. Potential toxicity of borax.	Localized drug delivery	[77]
Conductive hydrogel (PVA/CS/GO)	F/T cycle	1. Integration of electrical stimulation with drug delivery. 2. Dual effects: electronic drug release and tissue repair. 3. Low-voltage stimulation (2–5 V) enhances biological performance.	1. High concentrations of GO may raise concerns regarding long-term biocompatibility. 2. Potential cytotoxicity at low CS concentration.	Controlled transdermal drug delivery	[93]
rGO-PDA@ZIF-8/PVA/CS composite hydrogel	Bidirectional freezing method and phase separation technique	1. Excellent mechanical properties, low hemolysis rate, and water retention capabilities. 2. High biocompatibility and significant antibacterial effects against *E. coli* (99.1%) and *S. aureus* (99.0%). 3. Promoted wound healing effectively.	1. Slight decrease in wound healing area under 808 nm light irradiation due to higher temperature. 2. Incorporation of rGO-PDA@ZIF-8 slightly reduced water retention compared to PVA/CS alone.	Wound healing	[87]
PVA/CNT hydrogel	Freeze-casting-assisted compression annealing and salting-out (FCAS) strategy	1. Low hysteresis, good biocompatibility, and excellent mechanical properties (strength of 4.5 MPa and fatigue threshold of 1.5 kJ/m^2^). 2. High water content of 79.5%, comparable to natural ligaments. 3. Multifunctional properties (mechanical, electrical, and sensing).	1. Potential water loss during long-term use. 2. Limited exploration of long-term stability in physiological conditions.	Artificial ligaments Wearable sensors Flexible electronics Tissue engineering	[88]
Slippery PVA hydrogel	Salting-out-after-syneresis	1. Excellent optical transparency (98%). 2. Tribological coefficient down to 0.0081. 3. Excellent mechanical properties with tensile strength of 26.72 ± 1.05 MPa, modulus of 6.66 ± 0.29 MPa, and toughness of 55.21 ± 1.62 MJ/m^3^.	1. Potential reduction in hydration of surface networks with higher crystallinity.	Artificial biological soft tissues Wearable electronics	[78]

As research continues to advance, the potential for PVA-based hydrogels in tissue engineering is expected to expand, with ongoing investigations into their use in regenerative medicine, organ-on-a-chip technologies, and personalized tissue constructs. The versatility and tunability of PVA-based hydrogels position them as a cornerstone material in the developing innovative solutions for repairing and regenerating damaged tissues, ultimately contributing to the future of healthcare and regenerative therapies [88,105,106,111].

### 3.2. Smart and Responsive PVA-Based Hydrogels for Flexible Devices and Sensors

The emergence of smart materials has significantly reshaped the landscape of hydrogel applications, particularly in biomedical engineering, wearable technology, and soft robotics. Smart hydrogels, particularly those based on PVA, are designed to respond dynamically to external stimuli, including temperature, pH, light, and electric fields, thereby mimicking the adaptive behaviors of biological tissues. This responsiveness not only enhances their functionality but also expands their applicability across different fields, enabling innovations such as self-healing capabilities, shape memory effects, and improved mechanical properties [112,113,114,115,116]. In this section, we review the recent developments of smart and responsive PVA-based hydrogels for flexible devices and sensors, summarized in Table 2.

#### 3.2.1. Supercapacitor

Flexible energy storage devices face the dual challenges of devising toughness and maintaining a stable energy output during dynamic deformation. The incorporation of hydrogel polyelectrolytes in supercapacitors could promote the super-stretchability, compressibility, and ionic conductivity of supercapacitors [112,117,118,119]. For instance, Chen et al. introduced kosmotropic ions into PVA pre-hydrogels using a one-step solvent exchange strategy, replacing DMSO with water, leading to the self-assembly of PVA chains into a homogeneous network cross-linked by PVA crystalline domains and hydrophobic interactions. The resultant hydrogel demonstrated impressive mechanical properties with a tensile strength of 16.54 MPa, elongation at break of 1203%, and toughness of 111.21 MJ/m^3^, surpassing most reported hydrogels. Incorporating polyaniline electrodes into this supercapacitor yielded an areal specific capacitance of 156.50 mF/cm^2^ at 1.0 mA/cm^2^ and showed high mechanical resilience and retained stable energy output during deformation [60]. Peng et al. presented a novel physically cross-linked self-healing dual-network hydrogel electrolyte (PVA/Agar-EMIMBF_4_-Li_2_SO_4_) via one-pot physical cross-linking and F/T treatment. The addition of agar enhanced the tensile properties and flexibility of the hydrogel without significant capacity despite various bending angles. The dual-network hydrogel with ionic liquids exhibited excellent temperature tolerance between −30 °C and 80 °C and demonstrated a notable self-healing ability, recovering 80% of its initial state after five cycles [120]. Furthermore, Dong et al. fabricated a PAM/PVA/LiTFSI dual-network hydrogels by incorporating a sole ion source, resulting in remarkable electrical and mechanical properties (hyper-stretchability of 826% and high fracture stress of 162.2 kPa), viscoelasticity, and fatigue resistance due to synergistic intermolecular interactions. The addition of LiTFSI enhanced ionic conductivity (21.7 mS/cm) and anti-freeze properties. A supercapacitor with PAM/PVA/LiTFSI hydrogel electrolyte achieved an area-specific capacitance of 383.4 mF/cm^2^ at 0.5 mA/cm^2^, maintaining 90.35% capacitance after 10,000 cycles and excellent bending resistance [112]. These works offered strong potential for PVA-based hydrogels in wearable technology applications.

Inhibiting bacterial growth on hydrogels is essential for optimal operation of flexible supercapacitors [121,122]. Sun et al. reported an amphoteric sulfomethylated lignin/quaternized chitosan/PVA (SML/QCS/PVA) hydrogel electrolyte that showcased numerous charged groups from SML/QCS promoting KOH dissociation and forming ion transport channels that enhanced K^+^ and OH^−^ migration. This hydrogel achieved high ionic conductivity (46.64 mS/cm), tensile strain (927.32%), and compressive strain (85%) in ambient air, maintaining stable electrochemical performance under bending and heavy load. Additionally, these hydrogels exhibited notable antibacterial activity, with an inhibitory zone diameter above 37.0 mm for both *E. coli* and *S. aureus* [122].

Overall, these PVA-based hydrogels have emerged as promising materials for flexible supercapacitors, offering a unique combination of high ionic conductivity, excellent stretchability, and compressibility, as well as excellent antibacterial properties. Such innovations effectively address critical challenges in maintaining mechanical integrity and stable energy output under dynamic deformation, positioning PVA-based hydrogel supercapacitors as key components in the development of next-generation flexible and wearable energy storage devices.

#### 3.2.2. Flexible Sensor

When loaded with conductive substances, PVA-based hydrogels can function as conductive hydrogels, expanding their utility across numerous applications, such as supercapacitors and strain sensors. Their unique ability to conduct electricity while retaining the desirable properties of traditional hydrogels, such as flexibility and biocompatibility, makes them ideal for integration into advanced technological platforms and devices [20,112,113,123,124,125,126,127,128,129]. For instance, the PAM/PVA/LiTFSI dual-network hydrogels developed by Dong et al. can be integrated into wearable sensors, exhibiting fatigue resistance, rapid recovery after 400 stretches, high sensitivity (GF = 3.83 at 300–400%), and the capability to detect human motion in real time [112]. In the study by Patel et al., the conductivity of PVA/PDA@CDs hydrogels was significantly improved (from 0.57 to 1.46 mS/cm) due to the optimized distribution of conductive carbon dots (CDs) within the hydrogel matrix, facilitating electron, molecule, and ion transport. Additionally, these hydrogels demonstrated good sensitivity (GF = 3.8 at 180% strain) and were capable of real-time monitoring of human motions, highlighting their potential application in healthcare examinations [113].

To meet the need for fabricating flexible strain sensors with various shapes, Feng et al. combined thermosensitive κ-carrageenan (kC) with PVA and using borax as a cross-linking agent to create borax cross-linked PVA and kC conductive hydrogels (B-PVA/kC). Utilizing the thermal response behavior of kC enabled rapid sol–gel transition, allowing for injectable and 3D-printable hydrogels. The resulting B-PVA/kC hydrogels showed fast, sensitive, and accurate responses to movement (GF = 0.42 at 0–50% strain), along with good electrical conductivity, and maintained sensing properties (response time = 1 s; recovery time = 2 s) after F/T process [130,131]. 

The anti-freezing properties of PVA hydrogels open up more opportunities for their use in flexible devices [132]. Common strategies to improve anti-freezing properties include adding non-volatile organic solvents (such as ethylene glycol [133], glycerol [134,135], or mannitol [136]) to form hydrogen bonds with water, or incorporating ionic liquids [137], eutectic solvents [138,139], and inorganic salts [134,140] in water. For instance, Tao et al. prepared multifunctional silk fibroin/polyvinyl alcohol/glycerin/lithium chloride hydrogel (SPGL) using silk fibroin (SF), PVA, glycerin (Gly), and LiCl via F/T treatment. The Gly/H_2_O solvent system and LiCl inhibits ice formation and reduces water vapor pressure, providing anti-freezing capabilities (down to −70 °C) and anti-drying properties (only 17.4% weight loss after 30 days) of SPGL hydrogel [134]. However, the introduction of solvents may decrease the conductivity and mechanical properties of the hydrogels, limiting their application in sensors and other contact-based devices, such as bionic electronic skin and bio-oriented sensors [18,138].

Hydrogels with self-healing properties can autonomously repair damage and restore their original structure and functionality, making them highly valuable for applications in biomedical engineering, soft robotics, and flexible electronics [25,80,141,142]. PVA hydrogels possess excellent self-healing ability due to three main mechanisms: (i) dynamic hydrogen bonding, (ii) high chain mobility, and (iii) covalent cross-linking [17,25,113,114]. The self-healing ability is inversely related to the strength of the PVA-based hydrogel. Additionally, any parameter that slows down or hinders the mobility of the chain may adversely affect the self-healing ability. Other complementing strategies have been developed to enhance the self-healing properties [114,115]. For example, the MXene–polypyrrole/SF/PVA (PSDM) hydrogel demonstrated exceptional self-healing properties using dynamic borate ester bonds and reversible hydrogen bonding, confirmed to regain functionality regained after self-healing with a high efficiency (96.3%) even after four cutting/healing cycles [114]. Rong et al. achieved healing of PVA/poly(3,4-ethylenedioxythiophene):poly(styrenesulfonate) PVA/(PEDOT:PSS) hydrogels by contacting cut surfaces and a thermal cycle, achieving healing efficiencies above 85% while retaining conductivity, attributed to dynamic dissociation and re-association of crystalline domains and hydrogen bonds [67].

The conductive PVA-based hydrogel sensor shows potential for employment in the monitoring of human motion and might be incorporated into sensing preservation system to monitor ROS levels via real-time electronic signals. For example, Tian et al. presented an innovative zwitterionic PVA- and hydrogel-based (ZBA) sensing system for ultra-long hypothermic cell preservation up to 24 days. The system integrates a ROS-responsive zwitterionic hydrogel with a computer-controlled sensing and antioxidant delivery mechanism, allowing for real-time ROS monitoring and smart, on-demand antioxidant release to prevent oxidative cell damage during preservation. The 3D ECM-like environment provided by the hydrogel safeguards cells against anoikis, significantly extending the viable preservation duration and permitting gentle cell recovery compared to traditional methods, holding significant potential for therapeutic cells preservation, and durable cell-based living materials [143].

In general, these approaches offer promising routes for creating personalized, flexible sensors for applications in motion monitoring, health management, and human–computer interactions.

#### 3.2.3. Shape Memory Hydrogels

Shape memory hydrogels are smart materials capable of remembering and recovering their original shape after deformation, responding to stimuli such as temperature, pH, light, or electric fields. This exciting area of research has significant potential in biomedicine and soft robotics [144,145,146]. PVA-based hydrogels are particularly beneficial in these applications due to their good mechanical strength and elasticity, crucial for repeated deformation and recovery. They exhibit excellent shape memory capabilities through the formation of hydrogen bonds and crystalline regions that serve as physical cross-links. These cross-links can be melted and reformed, enabling the hydrogel to retain a deformed shape and revert to its original form upon stimulation. These properties make PVA-based hydrogels ideal candidates for shape memory applications [147,148].

**Figure 5:**
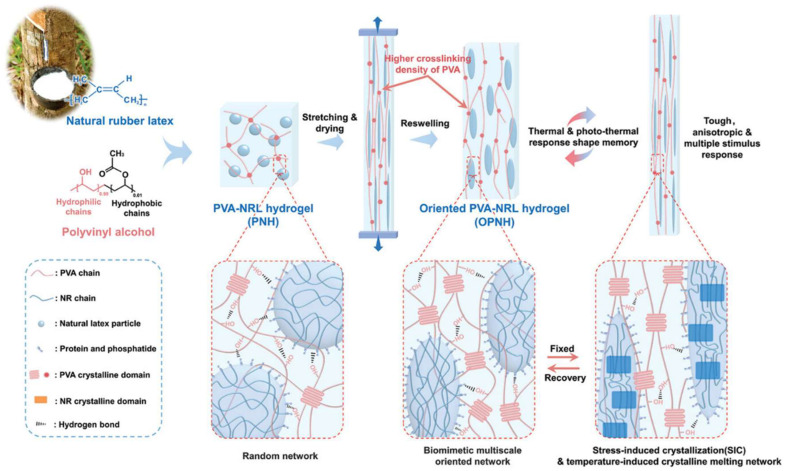
Schematic illustration of muscle-mimicking shape memory OPNH with microphase composite and oriented structures [147].

For instance, Wang et al. developed a triple shape memory PVA-based hydrogel by incorporating coordination bond between SA and Fe^3+^, the dynamic borate ester bonds, and hydrogen bonding of PVA [149]. Li et al. introduced an innovative shape memory hydrogel formulated as an oriented PVA/natural rubber latex hydrogel (OPNH, as shown in Figure 5). This novel material combines the SIC of natural rubber with a multiscale-oriented structure, achieved through a stretch-drying process. The resulting OPNH hydrogel demonstrated remarkable properties: outstanding mechanical performance (3.2 MPa), high shape fixity (≈80%) and shape recovery ratio (≈92%), high actuation strength (206 kPa) and working capacity (105 kJ/m^3^), extremely short response time (≈2 s), low response temperature (28 °C), and intelligent thermal response. Additionally, the hydrogel was able to hold muscle-like working capacity, lifting loads up to 372 times its own weight [147].

The integration of multifunctional small molecules such as melamine and tannic acid (TA) has also shown promise in enhancing the shape memory capabilities of hydrogels. These molecules form numerous and robust hydrogen bonds with hydroxyl groups in PVA chains, strengthening the structural integrity, and markedly improves their shape memory properties and overall performance [45,115,150]. For example, TA contains 25 hydroxyl and 10 carbonyl groups and forms hydrogen bonds with hydroxyl groups in PVA chains [45,150]. This network grants the PVA/TA supramolecular hydrogels their excellent shape memory properties. Remarkably, the deformed PVA/TA supramolecular hydrogels helix generally reformed its shape in 5 s after submerging it in water at 60 °C [150]. 

In summary, these findings open up new possibilities for designing advanced shape memory hydrogels with tailored properties for various applications in biomedical engineering and soft robotics.

#### 3.2.4. Actuator

PVA-based hydrogels, when modified to respond to various stimuli, provide excellent versatility for different applications. These hydrogels exhibit remarkable mechanical properties, making them suitable for robust actuator designs [147,151,152,153,154,155,156]. For instance, Deng et al. developed a high-performance artificial muscle hydrogel actuator consisting of microfibrillated cellulose (MFC), ionic liquid (IL), and PVA. The MFC-IL-PVA actuator features a high specific capacitance (225 mF/cm^2^), large bending strain (0.51%), peak displacement (7.02 mm at 0.25 V), excellent actuation endurance (99.1% holding rate for 3 h), and a broad frequency response (0.1–5 Hz). These characteristics are attributed to its high specific surface area, porosity, tunable mechanical properties, and strong ionic interactions. The MFC–IL–PVA actuator exhibits prospective applications such as bionic butterflies, bionic flytraps, and smart circuit switches [116]. Wang et al. developed an innovative bilayer hydrogel actuator with self-strengthening capabilities. The device consists of two layers of poly(N-isopropylacrylamide-*co*-hydroxymethyl acrylamide) P(NIPAM-*co*-NMA) hydrogel, each containing PVA nanocrystals of different sizes. This unique structure enables asymmetric swelling behavior, resulting in programmable thermal-responsive actuations. The incorporation of PVA crystallites not only enhances mechanical strength but also facilitates a self-reinforcement mechanism under cyclic loading. Upon repetitive tensile training, the hydrogel exhibits increased fracture stress and strain due to the anisotropic orientation and strain-induced crystallization of PVA domains. This biomimetic approach mimics the adaptive strengthening of biological tissues, addressing the challenge of maintaining durability in soft robotic applications [157]. 

While PVA-based hydrogel actuators show great promise, challenges remain, such as improving long-term stability, enhancing response speed, and increasing the magnitude of actuation. Future research is likely to focus on developing more sophisticated multi-responsive systems, improving the scalability of fabrication processes, and exploring novel applications in areas like soft robotics and tissue engineering.

#### 3.2.5. Triboelectric Nanogenerator (TENG)

The TENG is an ideal technology for a wide range of applications, particularly in the fields of wearable electronics, self-powered sensors, and sustainable energy harvesting [118,158,159]. In addition, hydrogel-based TENGs offer promising potential in wearable electronic devices. However, traditional hydrogel-based TENGs often suffer from insufficient electrical output and are not easily recyclable, leading to environmental concerns. Traditional hydrogels often exhibit poor electrical conductivity, limiting their effectiveness in energy harvesting applications [159,160,161,162]. It is still a challenge to develop new systems that can overcome these limitations while maintaining the benefits of hydrogel-based TENGs, such as biocompatibility and flexibility.

To address these issues, Pawisa Kanokpaka et al. reported a PVA-based hydrogel (GAH) incorporating glucose oxidase enzymes stabilized by β-cyclodextrin. The GAH undergoes conductivity changes in response to glucose concentrations, modulating the TENG output. The GAH-TENG exhibited excellent selectivity, stability, and self-healing properties. The GAH-TENG sensor successfully detected glucose levels in human sweat samples, highlighting its potential for diabetes management [158]. Patnam et al. synthesized an ionic conductive hydrogel by cross-linking PVA and CMC, followed by soaking in ionic solutions. This method creates ion-rich pores within the hydrogel, enhancing its conductivity and mechanical properties. By optimizing the CMC concentration and ionic solution type, the hydrogels achieved significantly improved single-electrode triboelectric nanogenerator (S-TENG) performance, with maximum voltage, current, and charge density outputs of 584 V, 25 μA, and 121 μC/cm^2^, respectively [159]. Zhou et al. reported an innovative development of a high-performance TENG based on LM/PVA hydrogel. The incorporation of liquid metal (LM) within a flexible PVA hydrogel matrix created a unique LP-TENG that synergistically combines triboelectrification, ion transport, and streaming vibration potential mechanisms. The innovative design offered several advantages: superior flexibility, biocompatibility, excellent electrical output (250 V open-circuit voltage; 4 μA short-circuit current), long-term durability, self-healing capability, and recyclability. The LP-TENG demonstrated multifunctionality in various applications, including human motion detection, energy harvesting, and human–machine interaction [160]. These approaches offer solutions to challenges in developing flexible and durable electrodes for wearable energy harvesting devices, offering a promising solution for sustainable and efficient mechanical energy harvesting applications.

Traditional hydrogel-based wearable sensors may exhibit low sensitivity and poor environmental resistance, hindering their practical applications [161,162,163]. Liu et al. fabricated a novel PVA-based nanocomposite hydrogel (MCGPP) with high ion conductivity that can function in harsh environments. The incorporation of MXene as ion-conducting microchannels and PSS as ion sources enabled directional transport of abundant free ions, significantly improving the sensitivity and mechanical–electrical conversion capabilities of the hydrogel. Additionally, the use of glycerol allowed for the hydrogel-based sensors to operate effectively at both low (−20 °C, GF = 3.37) and high (60 °C, GF = 3.62) temperatures. The resulting nanocomposite hydrogel exhibited excellent mechanical properties and superior piezoelectric and triboelectric performance over a wide temperature range [163].

**Table 2:** Summary of representative smart and responsive PVA-based hydrogels for flexible devices and sensors.

Hydrogel System	Methodology	Results	Limitation	Application	Ref.
PAM/PVA/LiTFSI hydrogel	One-step polymerization	1. High stretchability (826%), High fracture stress (162.2 kPa) 2. High ionic conductivity (21.7 mS/cm); area specific capacitance of 383.4 mF/cm^2^ 3. Good durability: 90.35% capacity retention after 10,000 cycles 4. Strain sensor with GF of 3.83 at 300–400% strain 5. High transparency (>90% transmittance)	1. Potential toxicity of chemical cross-linker	1. Flexible supercapacitors 2. Wearable sensors	[112]
SML/QCS/PVA	F/T method	1. High ionic conductivity: 46.64 mS/cm 2. Excellent mechanical flexibility and stretchability tensile strain = 927.32%, compressive strain = 85% 3. Excellent performance in flexible supercapacitor application: specific capacitance: 192.6 F·g⁻^1^; energy density: 45.2 Wh·kg⁻^1^; and maintained 86.1% capacitance retention after 10,000 cycles	1. Not extensively tested for performance at extreme temperatures and long-term stability in a variety of environments	1. Flexible wearable devices 2. Portable energy storage devices 3. Flexible supercapacitors	[122]
B-PVA/kC hydrogel	F/T cycles	1. Rapid sol–gel transition, good electrical conductivity 2. Good strain sensitivity (GF = 0.42 at 0–50% strain) 3. Enhanced mechanical properties after F/T cycles 4. Ability to rapidly form on curved surfaces or 3D-printed material	1. Dissolution in water over time (swelling behavior) 2. Limited long-term stability in aqueous environments	1. Flexible strain sensor	[130]
SPGL hydrogel	F/T cycles	1. Outstanding anti-freezing properties (<70 °C) and excellent anti-drying properties (17.4% weight loss after 30 days) 2. Recyclability (84.7% conductivity retention after remolding); strain sensors exhibited a GF = 2.18 and rapid response time = 0.2 s 3. Supercapacitors demonstrated high specific capacity (110.8 mF/cm^2^) and favorable cycle stability (88.5% capacitance retention after 10,000 cycles)	1. Relatively low ionic conductivity (52.63 mS /cm) compared to some other hydrogels	1. Flexible strain sensors 2. Supercapacitors	[134]
ZBA hydrogel	Boronic ester dynamic bond cross-linking	1. Longest reported shelf life for mammalian nucleated cells at refrigerated temperature 2. Dual protection against ROS overproduction and anoikis 3. Integration of smart hydrogel with computer-controlled system	1. Some cell death still occurred over extended preservation periods	1. Facilitation of cell-based clinical applications requiring extended storage or transport	[143]
OPNH	Physical cross-linking and orientation of the polymer chains	1. Muscle-inspired design with multiscale oriented structure 2. Shape memory function from stretch-induced crystallization of natural rubber 3. Excellent mechanical properties (3.2 MPa tensile strength) 4. High shape fixity (≈80%) and recovery ratio (≈92%). Fast response time (≈2 s) and low response temperature (28 °C) 5. High actuation strength (206 kPa) and working capacity (105 kJ/m^3^)	1. Complicated fabrication 2. CNT may not disperse evenly 3. Ensuring consistent stretch-drying and swelling is challenging	1. Smart biomimetic muscles 2. Multistimulus-responsive devices 3. Biomedical robotics	[147]
P(NIPAM-co-NMA)/PVA bilayer hydrogel	In situ photo polymerization and solvent exchange or F/T methods	1. Exhibited self-strengthening behavior, with tensile strength increasing from 29.6 kPa to 45.8 kPa and fracture strain increasing from 95% to 104% after 100 cycles of mechanical training. 2. Programmable transformations and excellent mechanical properties. 3. Novel strategy of using size-differentiated PVA crystallites for asymmetric structure	1. Potential damage from accumulated mechanical loading 2. Reduced mechanical strength at higher temperatures due to volume contraction	1. Intelligent soft robotics 2. Biomimetic hydrogel systems 3. Potential use in wound dressings, tissue engineering, strain sensors	[157]
LM/PVA hydrogel	Chemical cross-linking	1. High electrical performance: open circuit voltage of 250 V, short circuit current of 4 µA, and transferred charge of 120 nC 2. Excellent stability, recyclability, and self-healing capabilities. 3. Synergistic mechanism combining triboelectrification, ion transport, and streaming vibration potential (SVP)	1. Performance decreased with excessive LM content (>2.0 g) due to aggregation	1. Human motion detection 2. Handwriting recognition 3. Energy harvesting	[160]
MCGPP nanocomposite hydrogel	Assembly of MXene nanosheets and CNFs. The mixture was then cooled to form the hydrogel.	1. High sensitivity (gauge factor of 3.37 at −20 °C and 3.62 at 60 °C) 2. Excellent mechanical properties at low and high temperatures 3. High conductivity in harsh environments (−20 °C to 60 °C) 4. Fast response time (100 ms) and low detection limit (150 mg) 5. Good anti-freezing and moisturizing properties	1. Potential long-term stability issues not fully addressed	1. Self-powered electronics in harsh environments 2. Wearable sensors for human motion detection	[163]
S-TENG based on ionic conductive hydrogel	Cross-linking PVA and CMC, followed by soaking in ionic solutions	1. Maximum output: 584 V, 25 μA, and 120 μC/cm^2^. 2. Highly conductive, flexible, and stretchable 3. Stable performance over 15 days and long-term operation	1. Potential water evaporation over very long periods 2. Performance dependent on environmental conditions	1. Mechanical energy harvesting 2. Self-powered electronic displays 3. Smart touch sensors	[159]

Overall, these works collectively demonstrated the potential of self-powered, environmentally stable hydrogel-based sensors for flexible, wearable electronics in harsh environments. 

As researchers continue to explore the integration of functional materials and advanced fabrication techniques, devices based on PVA hydrogels are poised to play an important role in developing next-generation devices that can interact seamlessly with their environment, offering promising solutions for health monitoring, energy harvesting, and interactive applications. The ongoing advancements in these fields highlight the potential of smart hydrogels to revolutionize material science and engineering, opening the possibility of more sophisticated and wider applications in the future.

### 3.3. Environmental Treatment

PVA-based hydrogels are gaining attentions in environmental treatment due to their tunable properties and effectiveness in capturing and mitigating various contaminants. By implementing targeted optimization strategies, their effectiveness and applicability can be significantly enhanced [164,165,166,167,168,169]. This section provides a detailed analysis and explores optimization strategies to maximize their benefits.

#### 3.3.1. Solar Water Purification and Seawater Desalination

**Figure 6:**
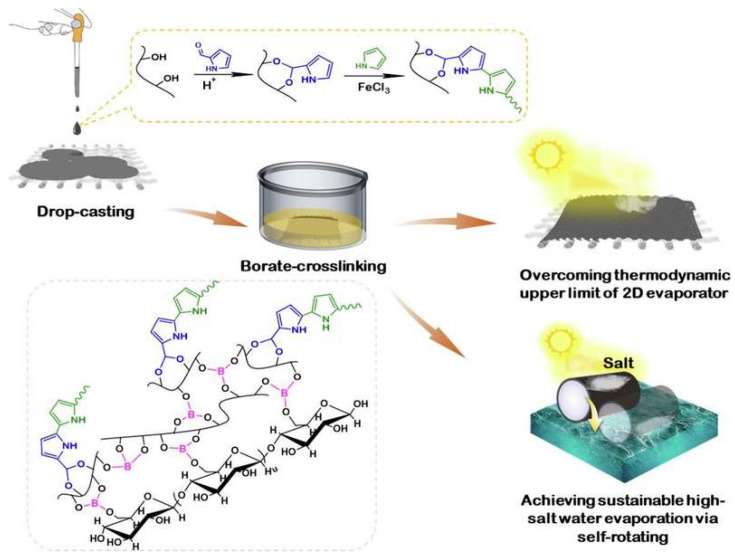
The fabrication, illustration, and working mechanism of a high-performance photothermal (PHF) hydrogel fabric for solar-driven interfacial evaporation [165].

The global water crisis necessitates innovative approaches for freshwater production from seawater and wastewater. Interfacial solar evaporation (ISE) has emerged as a promising solution due to its economic applicability, ease of fabrication, and environmental friendliness. ISE employs photothermal materials to harvest and convert solar energy efficiently, heating water molecules at the air–water interface to form vapor [164,170,171,172,173]. Hydrogel-based solar evaporators were demonstrated to achieve record-high evaporation rates (>3.0 kg. m^−2^. h^−1^) under one sun by tuning the interactions between polymer networks and water molecules. PVA-based hydrogels are ideal support matrices for these functional materials, thanks to their inherent chemical structure and physical properties [165,174,175,176]. For instance, in the work by Guo et al., a renewable hybrid hydrogel evaporator (HHE) combining konjac glucomannan (KGM), iron-based metal–organic framework (Fe-MOF)-derived solar absorbers, and PVA was introduced, processing several advantageous characteristics, including adequate water transport, effective water activation, and an anti-salt-fouling function, resulting in a high evaporation rate and effective performance (one sun (1 kW m⁻^2^) at 3.2 kg. m⁻^2^. h⁻^1^) under diverse conditions (high-salinity seawater of 330 g kg⁻^1^ and pH 2–14) [164]. Han et al. synthesized a high-performance photothermal (PHF) hydrogel fabric for solar-driven interfacial evaporation with PVA-decorated polypyrrole (PPy) nanoparticles that was immobilized by covalently onto cotton fabric through a simple drop-coating and borate cross-linking process (as shown in Figure 6) [165]. The resulting PHF hydrogel fabric exhibits excellent sunlight absorption (>92%) across a broad spectrum and surpasses the thermodynamic limit of planar evaporators, achieving evaporation rates of 1.62 kg.m^−2^.h^−1^ for pure water and 1.53 k kg.m^−2^.h^−1^ for pure water and 10 *wt%* NaCl solution, respectively. When configured into a 3D cylindrical structure, the evaporator demonstrates remarkable salt tolerance and self-renewal capabilities, maintaining stable performance in high-salt environments (20 *wt%* NaCl) for extended periods [165].

Generally, by employing the unique properties of PVA-based hydrogels and integrating them with advanced functional materials, innovative systems have been developed that achieve high evaporation rates, robust performance, and adaptability across various water sources and environmental conditions. These technologies offer significant potential in addressing the global water crisis by providing efficient, sustainable, and accessible solutions for producing freshwater from seawater and wastewater. These results provided an essential basis for the way of ultrahigh-speed solar seawater desalination under natural sunlight. However, the direct contact structure can lead to accelerated heat loss and a decreased light-to-heat conversion rate, posing challenges to their efficiency. Moreover, this design can limit the durability and usable lifespan of hydrogel materials, necessitating further optimization to enhance their performance and longevity [164,165,174,175,176,177,178].

#### 3.3.2. Efficiently Removal Pollutants from Water

The effective removal of heavy metal ions and dyes from wastewater has remained a significant challenge for decades [179,180]. The molecular structure of PVA processes a high density of hydroxyl (-OH) and acetate (-O-CO-CH3) groups, which endow PVA hydrogels with excellent capacity for adsorbing a wide range of contaminants, including anionic dyes, cationic dyes, and heavy metal ions, from wastewater [166,167,181,182,183,184]. For instance, Luo et al. developed a composite hydrogel (CBCS) consisting of PVA, CNT, an imidazolyl IL, and CS specifically for highly selective uranium adsorption from seawater. The hydroxyl groups, amino groups, and C=N bonds present on the CBCS surface play a direct role in uranium adsorption, while the dense pores contribute significantly to the process. Competitive adsorption experiments confirmed that CBCS exhibits exceptional selectivity for uranium, maintaining an adsorption rate of over 98% even after five cycles [166]. Gao et al. synthesized a novel green CS–PVA–diatomite hydrogel bead adsorbent through alkali solidification and tested for methylene blue (MB) removal from water. Structural analysis revealed the beads have a rough surface and high swelling capacity of 66.9 g/g. These hydrogels demonstrated an impressive maximum MB adsorption capacity of 414.70 mg/g, following the Freundlich isothermal and quasi-second-order kinetic models [167]. 

Despite extensive research on the removal of heavy metals and dyes individually, there remains a pressing need for a cost-efficient and straightforward method to treat wastewater by simultaneously removing various pollutants [167,185,186]. Addressing this challenge, Zhang et al. fabricated PVA/xanthan gum (XG) hydrogels via F/T treatment, which effectively adsorbed MB (27.39 mg/g) and lead ions (Pb^2+^, 17.07 mg/g) from a 50 mg/L solution. The adsorption followed the Langmuir isotherm and QFO models, indicating homogeneous physical adsorption, with predicted maximum adsorption capacities of 94.47 mg/g for MB and 58.50 mg/g for Pb^2+^. This innovative approach offers a cost-effective and eco-friendly solution for producing multifunctional PVA/XG hydrogels for efficient water treatment [182].

These developments in advanced PVA-based hydrogels demonstrate significant strides in wastewater treatment technologies [166,167,173,182,185]. These materials provide effective, selective, and reusable options for the removal of various contaminants, highlighting their potential for addressing some of the most pressing environmental challenges while being cost-effective and eco-friendly.

#### 3.3.3. Oil/Water Separation

PVA hydrogels are widely used in membrane fabrication, owing to their ease of modification, cost-effectiveness, and excellent membrane-forming properties. These attributes position PVA hydrogels as promising candidates for applications in oil/water separation treatments. Recent advancements have highlighted the effectiveness of PVA-based hydrogel membranes in achieving exceptional separation performance. Their ability to be easily tailored and their affordability make them an attractive option for developing efficient and sustainable solutions for oil/water separation challenges [85,168,187]. For instance, Zhu et al. introduced a novel squeezing coalescence demulsification (SCD) method for enhancing oil-in-water emulsion separation using PVA hydrogel nanofibrous membranes (PVA-HNM). The SCD process facilitates continuous high-flux separation (13,969 L/m^2^▪h) and achieves high efficiency (>95%) while overcoming membrane fouling issues [168]. To improve the mechanical strength and separation performance of emulsified oil and simultaneously remove heavy metal ions from complex oily wastewater, Chen et al. fabricated a PVA-CS-LDH hydrogel membrane by incorporating CS and layered double hydroxides (LDH) into a PVA hydrogel via F/T treatment and salting-out strategies. The hydrogel membrane exhibited high strength, excellent stretchability, an ideal 3D microstructure, and demonstrated high separation efficiency for emulsified oil (99.89%) and removal efficiency for Pb^2+^ ions (97.44%). This material overcomes the challenge of purifying emulsified oil and heavy metals simultaneously in complex oily wastewater, which is rarely achieved by existing engineering materials [85]. 

As research progresses, the development of PVA-based hydrogel is expected to play an important role in sustainable environmental management, offering eco-friendly and cost-effective solutions for efficient oil/water separation and contributing to the restoration of aquatic ecosystems.

#### 3.3.4. Air Purification

In addition to their applications in water treatment, PVA-based hydrogels can also serve as effective agents for air purification. Their high adsorption capacity allows for them to capture substantial amounts of particulate matter (PM) and associated pollutants. Furthermore, PVA-based hydrogels can be readily functionalized with photocatalysts such as TiO_2_, ZnO, CdS, and g-C_3_N_4_ for the removal of toxic pollutants, significantly enhancing their ability to degrade harmful organic compounds like polycyclic aromatic hydrocarbons (PAHs) under sunlight irradiation. The porous structure of hydrogels facilitates the efficient diffusion of contaminants, thus enhancing the photocatalytic degradation process [169,188,189]. 

For instance, Tandorn et al. introduced a biofunctionalized ZnO/PAM-PVA hydrogel composite material by using ZnO as photocatalyst. This hydrogel exhibited high photocatalytic activity for degrading PAHs in PM from various emission sources under sunlight irradiation. The composite effectively removed 95.15% of naphthalene (Nap), 79.49% of acenaphthylene (Acy), 88.79% of acenaphthene (Ace), and 95.60% of fluorene (Flu) from incense smoke. The synergistic effect of the adsorptive moist side and the photocatalyst-embedded side contributed to its efficiency. Moreover, its stability and recyclability were confirmed over multiple cycles, making it a promising candidate for practical applications in outdoor environments [169]. Wei et al. synthesized a highly efficient, environmentally friendly dust suppressant (PVA-XG-PAA/SDBS hydrogel) based on the hydrogen bonding interactions between PVA, XG, and acrylic acid (AA) polymers. The suppressant demonstrated low surface tension (30 mN/m), suitable viscosity (45 mPa·s), and high compression strength (126 kPa) for solidified coal dust, and could reach a 34% degradation rate after 40 days in soil, highlighting its eco-friendly nature. These properties make PVA-XG-PAA/SDBS an effective and sustainable solution for dust control in coal mining, transportation, and storage [189].

In summary, the multifunctionality of PVA-based hydrogels, combined with their environmental friendliness and cost-effectiveness, makes them a promising material in advancing sustainable technologies across various industries.

### 3.4. Civil Engineering

The use of hydrogels, including PVA-based hydrogel, in concrete has been well researched and has shown promising outcomes in enhancing the performance in civil engineering [84,190,191,192].

#### 3.4.1. Improvement of Freezing/Thawing Resistance in Concrete

Conventional concrete is prone to damage from repeated cycles of freezing and thawing, leading to internal stresses, cracking, and eventual structural failure. Controlling the free movement of water and mitigating internal pressures during freezing is critical [193,194]. Hydrogels, with their unique water retention and temperature-responsive properties, present a promising avenue of investigation [84,191,192]. 

Motivated by the need to improve the durability of concrete in cold regions, Wu et al. developed a smart, eco-friendly cellulose/PVA hydrogel that can be easily incorporated into cement mixes. The hydrogels form a 3D network structure within the concrete, which retains water during freezing and releases it during thawing, thus optimizing the internal structure of the cement paste. Moreover, the hydrogels effectively reduce osmotic pressure, preventing crack propagation. The system demonstrated improved compressive strength retention after F/T cycles, a modified microstructure with increased fine pores, and improved overall durability. Additionally, the hydrogel promoted early cement hydration and offered a sustainable solution for extending the life of concrete structures in harsh environments. The research represents a promising method of using advanced materials science to address a persistent challenge in concrete technology, potentially revolutionizing concrete performance in cold regions [84].

#### 3.4.2. High Performance Concrete

Traditional cement-based materials are inherent limited by their quasi-brittleness and low toughness, primarily due to the disordered distribution of hydration products and pore structures [195,196]. Efforts to enhance cement properties, such as the incorporation of reinforcing materials or the modification of cement slurry properties, have only shown moderate improvements, typically limited to no more than a doubling in ductility and toughness. Additionally, these methods often adversely affect the cohesiveness of the slurry preparation process and potentially reduce compressive strength [196,197,198].

**Figure 7:**
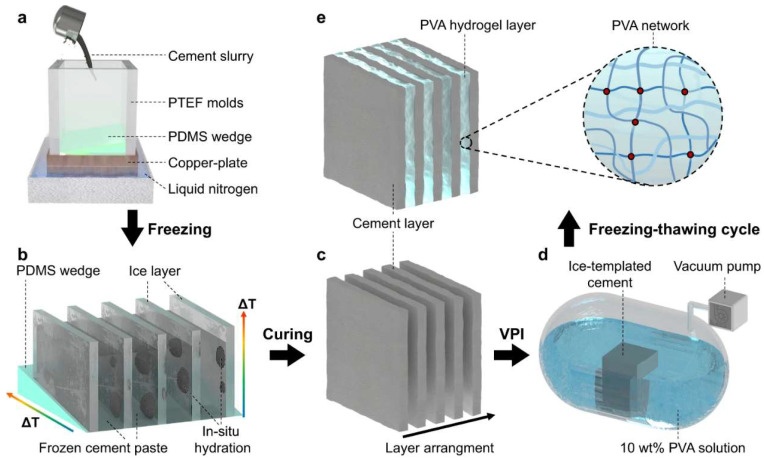
Schematic illustrations of the fabrication process of the cement–hydrogel composite. (**a**) The mixing of cement slurries in the mold with internal dimensions of 50 × 50 × 50 mm. (**b**) Cement slurries solidify into ice layers by a bidirectional freezing gradient both vertically and horizontally. Cement particles are squeezed between ice layers and slowly hydrate in situ. (**c**) Cement particles hydrate into order layers during a thawing and curing period with a thickness of 10–100 μm. (**d**) The PVA solution was filled into the pores between neighboring cement sheets by VPI (vacuum pressure impregnating). (**e**) PVA hydrogels were formed between the cement lamellae after 2~3 freezing/thawing cycles [199].

Inspired by natural strengthening mechanisms observed in nacre (mollusk shells), Chen et al. innovatively combined a simplified ice-template method to create an ordered biomimetic cement skeleton with in situ polymerization of PVA hydrogels in the interlayer spaces (as shown in Figure 7). This groundbreaking approach resulted in a remarkable 175-fold increase in toughness compared to pure cement paste while also more than doubling the flexural strength. The composite material demonstrated multifunctional properties, including low density (1.44 g/cm^3^, 40% reduction), thermal insulation (0.21 W/m.K, 80% reduction), and self-healing capabilities. Molecular dynamics simulations and finite element analysis revealed the underlying toughening mechanisms, which involve hydrogel stretching and crack deflection. These insights provide a deeper understanding of how such composites can achieve superior mechanical performance. This innovative material shows promise for applications in earthquake-resistant structures, energy-efficient buildings, and durable infrastructure, potentially revolutionizing construction materials [199].

#### 3.4.3. Waterproofing Materials 

Waterproofing systems are crucial in underground construction, particularly in tunnels and basements. However, existing waterproofing materials, such as sprayed concrete and waterproof membranes, still have limitations and often suffer from long-term issues such as leakage and high maintenance costs. Traditional hydrogel membranes lack sufficient strength and self-healing capabilities, making them susceptible to cracking under load and ineffective in preventing water ingress under hydrostatic pressure [200,201,202].

There is pressing demand for advanced waterproofing materials that offer both robust mechanical properties, self-healing capabilities, and long-term resilience against water ingress under various pressure conditions. Lee et al. developed a novel hydrogel by combining magnesium acrylate (CA-Mg_2_) with PVA via F/T treatment. The hydrogels were cross-linked by coordination bonds from CA-Mg_2_ and hydrogen bonds from PVA, resulting in improved mechanical properties and rapid self-healing capabilities. This material showed significantly improved fracture toughness and elongation compared to CA-Mg_2_ hydrogels alone, with a self-healing efficiency of up to 99.9% within 3 h. When applied as a shot-membrane waterproofing material, it demonstrated the ability to self-heal under water pressure and repair cracks repeatedly, addressing the challenges of maintaining and repairing waterproof layers in construction engineering [202].

#### 3.4.4. Flame-Retardant Materials

As fire-related incidents continue to rise in residential and industrial areas, there is growing demand for effective fire-resistant materials. Conventional halogen-based flame retardants pose significant environmental and health hazards, prompting the development of safer alternatives. PVA-based hydrogels have emerged as promising candidates due to their water retention capabilities, which can help suppress fires [203,204].

To create innovative fire suppressants that are both effective and environmentally responsible, Zhao et al. fabricated a novel fire-retardant PVA-based hydrogel with XG and lignin nanoparticles (LNPs) as eco-friendly additives. The use of biodegradable biopolymers and lignin nanoparticles enhanced the fire-retardant properties of PVA hydrogels. The hydrogels exhibited improved thermal stability, char formation, and fire resistance, as demonstrated by increased limiting oxygen index (LOI) values and reduced heat release rates. The hydrogels also showed good water absorption and retention capabilities, making them suitable for fire-resistant coatings. This green approach offers a promising alternative to traditional flame retardants, with potential applications in fire-resistant textiles and protective gear [204].

The use of PVA-based hydrogels in civil engineering, particularly concrete technology, holds great promise in addressing challenges in the construction industry. These advancements enhance the longevity and safety of structures while promoting sustainable practices by reducing repair needs. Future innovations may include integrating PVA hydrogels with advanced materials for multifunctional concrete composites that offer improved thermal insulation, electromagnetic shielding, or energy harvesting. To fully harness their potential, research should focus on optimizing long-term performance under diverse conditions, scaling production for large applications, and conducting lifecycle assessments for economic and environmental viability. As challenges are addressed in the coming future, PVA-based hydrogels are set to revolutionize construction technology, contributing to more durable, sustainable, and resilient infrastructure.

### 3.5. Other Emerging Application of PVA-Based Hydrogels

PVA-based hydrogels have gained significant attention in various emerging applications due to their unique tunability properties, opening new possibilities for solving complex challenges across multiple fields.

Water vapor, a ubiquitous component of the Earth’s atmosphere, plays a key role in the global energy balance and the hydrological cycle [205,206]. Guo et al. addressed the challenge of harnessing electricity from ubiquitous water vapor by presenting an innovative bilayer polymer system for efficient and self-sustaining moisture electricity generation. The device consists of a hydrophobic porous P(VdF-HFP) top layer and a hygroscopic ionic PVA hydrogel bottom layer. The top layer prevents excessive evaporation during the daytime and accelerate moisture absorption at night. This is complemented by the LiCl-enhanced hydrogel bottom layer, which significantly improves moisture retention and enhances ion transport. This design allows for continuous power output even under fluctuating outdoor conditions, with a single 1 cm^2^ device unit producing ~0.88 V and ~306 μA, achieving a maximum power density of 51 μW/cm^2^ at 25 °C and 70% RH. The system demonstrated excellent environmental adaptability, maintaining stable output for over 6 days in outdoor tests, demonstrating its potential for sustainable energy harvesting in various real-world applications, offering a novel solution to pressing global energy challenges [205].

PVA-based hydrogels cross-linked with boric acid (BA) have been developed as a potential radiation shielding material for use in space applications [81,207,208]. The combination of PVA with BA in hydrogels results in enhanced mechanical properties and water retention compared to pure PVA hydrogels, while maintaining comparable radiation shielding effectiveness to liquid water, especially at lower thicknesses. These PVA/BA hydrogels low modulus showed promising potential for integration into spacesuits as thin, flexible layers offering reduced weight and improved protection compared to current materials. The findings of the study illustrate the potential of PVA/BA hydrogels for use in space applications, where both radiation shielding and flexibility are essential [207].

**Figure 8:**
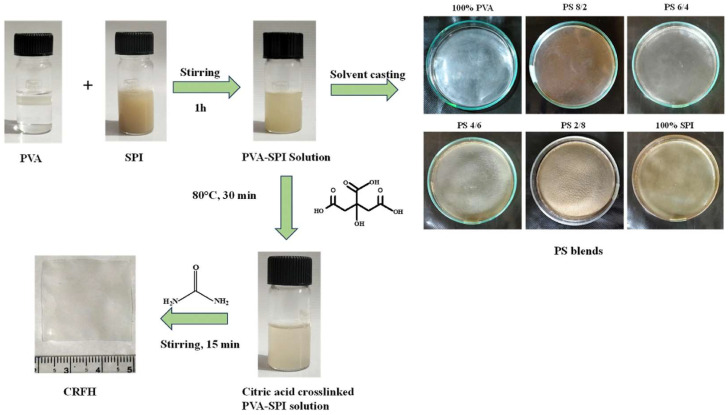
Illustrations for the fabrication of PVA–soy protein isolate blends (PS) and controlled release fertilizer hydrogel (CRFH) [209].

In agriculture, PVA-based hydrogels have been used for water retention in crops, moisture conservation, soilless cultivation, seed coating, and controlled release of fertilizers. The porous structure of these materials promotes the availability of oxygen and water in the root system, stimulating physiological parameters of the plant and promoting its growth [209,210,211,212]. For instance, a hydrogel (CRFH, as shown in Figure 8) based on PVA and soy protein isolate (SPI) cross-linked with citric acid not only provided controlled release of urea (74.1% over 28 days) but also gradually released nutrients from the soy protein matrix. Additionally, the CRFH demonstrated excellent water retention capacity, enhancing drought resistance in plants [209]. By adjusting the poly(acrylic acid) (PAA) content, Seeponkai et al. optimized the mesh size, water uptake, mechanical properties and ion permeability of the PVA-based hydrogel membranes for plant growth, as demonstrated by successful tomato seed germination experiments [210]. Fabian et al. created a novel biopolymer hydrogel using acid whey, cellulose derivatives, and PVA, aiming to improve water retention in soil and enhance plant growth. The addition of PVA to the hydrogel composition enhanced its stability and prolonged its degradation time in soil. The resulting hydrogel demonstrated excellent swelling properties, improved water retention in soil by up to 19%, and positively affected the growth of Brassica napus and Sinapsis alba [212]. The studies provided valuable insights into designing PVA-based hydrogel with tailored properties for specific agricultural applications.

Conventional wet cleaning techniques frequently fail to offer the requisite precision, control, and selectivity necessary for the delicate conservation of fragile paper artworks, thereby increasing the risk of damage or unintended alterations. In contrast, hydrogel-based approaches have emerged as innovative tools that effectively address these limitations [213,214]. By fully using the distinctive characteristics of PVA and its derivative, conservators can attain more effective and gentle cleaning of paper artworks, thereby ensuring their structural integrity and longevity [215,216]. For instance, Mazzuca et al. developed PVA-based hydrogels cross-linked with telechelic PVA (tel-PVA), constituting a substantial advancement in this field. The hydrogels are designed to address challenges associated with conventional cleaning techniques, including excessive water uptake, fiber swelling, and the uncontrolled spread of degradation products. PVA-based hydrogels offer several advantages, including non-perishability and long-term storage. Furthermore, the hydrogels possessed tunable properties through adjustable PVA/tel-PVA ratios. In addition, the hydrogels exhibited high water retention, minimizing potential damage to fragile paper. The hydrogels effectively removed dust, degradation byproducts, and patinas [215]. These findings make PVA-based hydrogels an invaluable tool for art conservators seeking to protect and preserve the cultural heritage embedded in paper-based artifacts.

In summary, the emerging applications of PVA-based hydrogels demonstrate their remarkable multifunctionality and potential to address complex challenges in various fields. From harnessing energy from atmospheric water vapor to preserving delicate artworks, these hydrogels prove invaluable in areas previously unexplored. The ability of PVA hydrogels to be tailored for specific applications, such as moisture electricity generation and art conservation, highlights their adaptability and the ongoing innovation in hydrogel technology.

## 4. Conclusions and Perspectives

The advancements in polyvinyl alcohol (PVA)-based hydrogels have demonstrated significant potential in addressing the challenges faced by traditional hydrogels. PVA-based hydrogels are distinguished by their unique properties, including excellent biocompatibility, tunable mechanical characteristics, and the ability to form stable three-dimensional networks. These attributes position them as multifunctional materials suitable for a wide range of applications including in the biomedical, environmental, and industrial fields.

Recent research has highlighted the effectiveness of innovative manufacturing techniques, such as freeze/thaw cycles, chemical cross-linking, and the incorporation of various additives, which have led to the development of PVA-based hydrogels with enhanced mechanical strength, elasticity, and responsiveness to external stimuli. These improvements have expanded the applicability of PVA-based hydrogels in critical areas such as drug delivery systems, wound healing, tissue engineering, and flexible devices. The integration of nanoparticles and the development of composite structures have further enhanced the functionality of PVA-based hydrogels, enabling their use in advanced applications such as smart sensors, actuators, and energy storage devices.

Despite these advancements, several challenges remain. The mechanical strength and stability of PVA-based hydrogels under varying environmental conditions remain critical areas of concern, particularly in applications where durability is paramount. Additionally, the biocompatibility of chemical cross-linkers and the potential for toxicity in certain applications necessitate the exploration of alternative, non-toxic cross-linking methods. Furthermore, the scalability of manufacturing processes and the economic feasibility of advanced hydrogel systems must be addressed to facilitate their widespread adoption in clinical and industrial settings.

Looking ahead, the future of PVA-based hydrogels appears promising. Ongoing research is likely to focus on the integration of smart functionalities, such as self-healing capabilities, antimicrobial properties, and enhanced multi-responsiveness to physiological and chemical conditions. The incorporation of nanocomposite technologies and the use of natural, biodegradable materials; the application of 3D printing techniques; as well as the integration of artificial intelligence and machine learning are also expected to play a crucial role in the development of next-generation hydrogels. These advancements will not only improve the performance of PVA hydrogels but also align with the growing demand for sustainable and environmentally friendly materials.

In conclusion, PVA-based hydrogels represent a significant advancement in hydrogel technology, offering innovative solutions to existing limitations while opening new avenues for research and application. The versatility of PVA-based hydrogels, combined with their biocompatibility and tunable properties, positions them at the forefront of materials science innovation. As research progresses, we can expect to see the development of increasingly sophisticated PVA-based hydrogel systems that blur the boundaries between synthetic materials and living tissues, potentially revolutionizing fields such as regenerative medicine, environmental science, and advanced manufacturing.

Continued exploration into novel fabrication methods, material combinations, and functional enhancements will be essential in realizing the full potential of PVA-based hydrogels. Interdisciplinary collaboration between materials scientists, biologists, engineers, and clinicians will be crucial in addressing the remaining challenges and pushing the boundaries of what is possible with these remarkable materials. Ultimately, these efforts will contribute to the development of materials that can meet the demanding requirements of modern healthcare, environmental sustainability, and industrial innovation, paving the way for a future in which intelligent hydrogels play an integral role in addressing some of society’s most pressing challenges.

## Abbreviations

Polyvinyl alcohol (PVA); sodium alginate (SA); hydroxyethyl cellulose (HEC); montmorillonite (MMT); zinc ions (Zn^2+^); freeze/thaw (F/T); dimethyl sulfoxide (DMSO); electron beam irradiation (EBI); chitosan (CS); polyvinylpyrrolidone (PVP); carbon–carbon (C-C) bonds; glutaraldehyde (GA); ammonium persulfate (APS); polyacrylamide (PAAm, PAM); hydroxypropyl chitosan (HPCS); oxidized sodium alginate (OSA); graphene oxide (GO); poly(2-methoxyethyl acrylate) (PMEA); sodium fluorescein (NaFL); vascular endothelial growth factor (VEGF); basic fibroblast growth factor (FGF); dopamine (DA); extracellular matrix (ECM); carbon nanotubes (CNT); reactive oxygen species (ROS); 1-ethyl-3-methylimidazolium tetrafluoroborate (EMIMBF4); sulfomethylated lignin (SML); quaternized chitosan (QCS); carbon dots (CDs); κ-carrageenan (kC); silk fibroin (SF); gauge factor (GF); glycerin (Gly); lithium chloride (LiCl); silk fibroin/polyvinyl alcohol/glycerin/lithium chloride hydrogel (SPGL); poly(3,4-ethylenedioxythiophene):poly(styrenesulfonate) (FEDOT:PSS); phosphate-buffered saline (PBS); carboxybetaine methacrylate (CBMA); 3-methacrylamidophenylboronic acid (MPBA); stretch-induced crystallization (SIC); poly(N-isopropylacrylamide-*co*-hydroxymethylacrylamide) (P(NIPAM-*co*-NMA); liquid metal (LM); triboelectric nanogenerator (TENG); eutectic gallium-indium-based liquid metal (EGaIn); MXene−CNF−glycerol−PSS−PVA (MCGPP); poly(sodium 4-styrenesulfonate) (PSS); cellulose nanofibrils (CNFs); carboxymethyl cellulose (CMC); glucose adaptive hydrogel (GAH); glucose oxidase (GOx); β-cyclodextrin (β-CD); functionalized multiwalled carbon nanotubes (f-MWCNTs); polyvinyl alcohol/poly(2-methoxyethylacrylate) (PVA/PMEA); U.S. Food and Drug Administration (FDA); reduced graphene oxide (rGO); bilirubin/morin nanoparticles (BMn); poly(N-isopropylacrylamide) (poly(NIPAM)); hydroxymethyl acrylamide (NMA); microfibrillated cellulose (MFC); ionic liquid (IL); konjac glucomannan (KGM), iron-based metal–organic framework (Fe-MOF); polypyrrole (PPy).
